# A Quantitative Model of Sporadic Axonal Degeneration in the *Drosophila* Visual System

**DOI:** 10.1523/JNEUROSCI.2115-21.2022

**Published:** 2022-06-15

**Authors:** Mélisande Richard, Karolína Doubková, Yohei Nitta, Hiroki Kawai, Atsushi Sugie, Gaia Tavosanis

**Affiliations:** ^1^Deutsches Zentrum für Neurodegenerative Erkrankungen e. V., 53127 Bonn, Germany; ^2^Brain Research Institute, Niigata University, Niigata 951-8585, Japan; ^3^LPIXEL, Tokyo 100-0004, Japan; ^4^Life & Medical Sciences Institute (LIMES), University of Bonn, 53115 Bonn, Germany

**Keywords:** axon, *Drosophila*, neurodegeneration, neurotransmission, sporadic, synapse

## Abstract

In human neurodegenerative diseases, neurons undergo axonal degeneration months to years before they die. Here, we developed a system modeling early degenerative events in *Drosophila* adult photoreceptor cells. Thanks to the stereotypy of their axonal projections, this system delivers quantitative data on sporadic and progressive axonal degeneration of photoreceptor cells. Using this method, we show that exposure of adult female flies to a constant light stimulation for several days overcomes the intrinsic resilience of R7 photoreceptors and leads to progressive axonal degeneration. This was not associated with apoptosis. We furthermore provide evidence that loss of synaptic integrity between R7 and a postsynaptic partner preceded axonal degeneration, thus recapitulating features of human neurodegenerative diseases. Finally, our experiments uncovered a role of postsynaptic partners of R7 to initiate degeneration, suggesting that postsynaptic cells signal back to the photoreceptor to maintain axonal structure. This model can be used to dissect cellular and circuit mechanisms involved in the early events of axonal degeneration, allowing for a better understanding of how neurons cope with stress and lose their resilience capacities.

**SIGNIFICANCE STATEMENT** Neurons can be active and functional for several years. In the course of aging and in disease conditions leading to neurodegeneration, subsets of neurons lose their resilience and start dying. What initiates this turning point at the cellular level is not clear. Here, we developed a model allowing to systematically describe this phase. The loss of synapses and axons represents an early and functionally relevant event toward degeneration. Using the ordered distribution of *Drosophila* photoreceptor axon terminals, we assembled a system to study sporadic initiation of axon loss and delineated a role for non-cell-autonomous activity regulation in the initiation of axon degeneration. This work will help shed light on key steps in the etiology of nonfamilial cases of neurodegenerative diseases.

## Introduction

A remarkable property of neurons is their resilience. Although most cells in the human body undergo frequent turnover, neurons in the central and peripheral nervous system can remain active and functional for decades (Sender and Milo, 2021). Nonetheless, in neurodegenerative conditions and during aging, this resilience is overcome, and progressive degeneration starts (Erkkinen et al., 2018; Hou et al., 2019). In this context, the initiation of neurodegeneration at the cellular level is represented by the shift from a resilient state to an unstable condition that ultimately leads to neuronal death. A small fraction of patients who develop commonly occurring neurodegenerative diseases (NDs), such as Alzheimer's disease (AD) or Parkinson's disease (PD), display a familial predisposition (Brown, 1997; Tang and Gershon, 2003; Schiesling et al., 2008). Nonetheless, the vast majority of cases of neurodegenerative diseases do not have a clear familial history; they are thought to be sporadic, whereby aging represents a major risk factor (Erkkinen et al., 2018; Hou et al., 2019). Although developmental defects could underlie the onset of sporadic cases, a large body of evidence suggests that imbalanced neuronal activity leads to loss of neuronal resilience and triggers neurodegeneration (Palop et al., 2007; Busche and Konnerth, 2016; Palop and Mucke, 2016; Arendt et al., 2017; Keogh et al., 2018; Sosulina et al., 2021).

At the cellular level, the process of neuronal degeneration starts with the gradual loss of axons and dendrites (Adalbert and Coleman, 2013; Kweon et al., 2017). Axonal loss precedes degeneration of neuronal cell bodies by months to years, through a process of retrograde degeneration (Cavanagh, 1979; Neukomm and Freeman, 2014; Tagliaferro and Burke, 2016). Importantly, synapse loss precedes cognitive decline in many neurodegenerative diseases and is closely correlated with pathology progression (Henstridge et al., 2018; Colom-Cadena et al., 2020).

Invertebrate models, including *Drosophila*, have greatly contributed to our understanding of neurodegenerative disorders, allowing for the identification and characterization of involved molecular factors (Lu and Vogel, 2009; Neukomm and Freeman, 2014; McGurk et al., 2015; Vanhauwaert and Verstreken, 2015; Wu and Lloyd, 2015; Şentürk and Bellen, 2018; Bolus et al., 2020). These models often rely on genetic manipulation of *Drosophila* orthologs of human neurodegenerative disease proteins or on transgenic expression of human proteins linked to familial neurodegenerative diseases. However, a quantitative model for sporadic initiation of axonal degeneration is missing in this highly tractable model organism.

Here, we sought to develop a system in which axons of wild-type animals start to degenerate sparsely and reliably in defined conditions, with the aim of elucidating the mechanisms that trigger the switch from resilience to vulnerability of axons. For this purpose, we have developed a new fly model, combining important characteristics. First, we chose to work with photoreceptors (PRCs) to allow for easy manipulation of activity by modulating the intensity or time exposure to light. Second, well-defined numbers of PRCs project their axon to higher visual processing centers. In particular, the R7 subset of PRCs projects to the medulla, where R7 axons distribute in highly ordered maps. The loss of individual axons is readily apparent in this organization. Therefore, the initial cases of axon loss can be easily identified. Third, the circuit formed downstream of PRCs is described in detail at the electron microscopy level, and sophisticated genetic tools allow the manipulation of individual relevant cell populations (Morante and Desplan, 2008; Takemura et al., 2008, 2013).

We show that exposure of flies to a constant light stimulation leads to a progressive light-dependent axonal degeneration in R7 PRCs, which was not accompanied by apoptosis. These axons slowly accumulated reversible damage up to a point of no return, which was reached at a different time point for individual axons and after which degeneration was initiated. Importantly, synaptic integrity between R7 and a medullar postsynaptic partner was lost before axons degenerated, thus recapitulating early features of human neurodegenerative diseases. Finally, our data delineate the role of postsynaptic partners of R7 in the initiation of presynaptic axon degeneration.

## Materials and Methods

### Fly strains and light treatment

Flies were kept on standard medium in an incubator at 25°C and 60% humidity (CLF PlantClimatics) and allowed to develop with a 12 h light/dark (LD) cycle. After eclosion, female flies were collected within 24 h and kept in food vials at 25°C and 60% humidity. For LD treatment, flies were illuminated with a 12 h light/dark cycle. We noticed that a light intensity up to 10,000 lux in the LD cycle was not deleterious to axons (see Fig. 3*I*,*I'*,*J*,*K*). For constant light (LL) conditions, we used an LED light source (6 W, warm white, Heitronic) and placed the flies in food vials at a light intensity of 10,000 lux, the intensity being measured at the top of the food in the vials and in the direction of the light source (Volt Craft MS-1300 photometer, Conrad). For constant darkness (DD), vials were wrapped in aluminum foil and kept in the same conditions as for LL.

All genotypes used are listed in Table 1. The following lines were obtained from the Bloomington Drosophila Stock Center: *GMRwhite RNAi* (stock #32067), *GMR-Gal4* (stock #1104), *UAS-tubulinGFP* (stock #7374), *UAS-p35* (stock #5072), *lexAop-spGFP1-10, UAS-CD4spGFP11* (stock #64315), *UAS-Dcr2* (I) (stock #24648), *UAS-Dcr2* (II) (stock #24650), *UAS-Dcr2* (III) (stock #24651), *tubGal80^ts^* (stock #7018 and 7019), *w* (stock #3605), *ort^5^* (stock #29637), *UAS-CD8GFP* (stock #32186), and *UAS-syd-1 RNAi* (stock #6447). *UAS-nrx RNAi* (stock #36328) and *UAS-lip-*α *RNAi* (#106588) were obtained from Vienna Drosophila Resource Center. The *40D-UAS* line was used as a transgenic control and was also obtained from the Vienna Drosophila Resource Center (stock #60101). *Rh4-lexA* was described in Berger-Müller et al. (2013). The *ort^1^* (stock #106217) was obtained from the Kyoto Stock Center. The *ortc2b-Gal4* was a gift from from Chi-hon Lee (Gao et al., 2008), *UAS-shi^ts^* was from Prof. Michael Pankratz (Kitamoto, 2001), and *UAS-dTrpA1* was from Prof. H. Tanimoto (Hamada et al., 2008). For *shi^ts^* experiments, control and *UAS-shi^ts^*-expressing flies were raised in LD at 20°C. After eclosion, adults were shifted to LL at 29°C. For *TrpA1* experiments, flies were raised in LD at 20°C, and adults were kept at 29°C (test) or 20°C (control) in DD.

**Table 1.** Genotypes of flies used in the main figures

Figure	Genotype
Figure 1	*GMRwhite RNAi/w;GMR-Gal4/*+*;UAS-tubulinGFP/*+
Figure 2*A* (top row)	*w;40D-UAS/CyO;UAS-Dcr2/TM6B* (transgene a) *w;UAS-Dcr2/CyO;UAS-syd-1 RNAi/TM6B* (transgene b) *w;UAS-nrx RNAi/CyO;UAS-Dcr2/TM6B* (transgene c) *w;UAS-lip-*α *RNAi/CyO;UAS-Dcr2/TM6B* (transgene d)
Figure 2*A* (bottom row)	*GMRwhite RNAi/w;GMR-Gal4/40D-UAS;UAS-tubGFP/UAS-Dcr2* (transgene a) *GMRwhite RNAi/w;GMR-Gal4/UAS-Dcr2;UAS-tubGFP/UAS-syd-1 RNAi* (transgene b) *GMRwhite RNAi/w;GMR-Gal4/UAS-nrx RNAi;UAS-tubGFP/UAS-Dcr2* (transgene c) *GMRwhite RNAi/w;GMR-Gal4/ UAS-lip-*α *RNAi;UAS-tubGFP/UAS-Dcr2* (transgene d)
Figure 3	*GMRwhite RNAi/w; GMR-Gal4/*+*;UAS-tubulinGFP/*+
Figure 4	females:*GMRwhite RNAi/w; GMR-Gal4/*+*;UAS-tubulinGFP/*+ males: *GMRwhite RNAi/Y; GMR-Gal4 UAS-tubulinGFP*/+
Figure 5	*GMRwhite RNAi/w; GMR-Gal4/*+*;UAS-tubulinGFP/*+
Figure 6	*GMRwhite RNAi/w; GMR-Gal4/*+*;UAS-tubulinGFP/*+
Figure 7	control: *GMRwhite RNAi/w; GMR-Gal4 UAS-tubulinGFP/40D-UAS* p35: *GMRwhite RNAi/w; GMR-Gal4 UAS-tubulinGFP/UAS-p35*
Figure 8 *A–C*	control: *GMRwhite RNAi/w; GMR-Gal4 UAS-tubulinGFP/UAS-CD8GFP* *TrpA1*: *GMRwhite RNAi/w; GMR-Gal4 UAS-tubulinGFP/UAS-TrpA1*
Figure 8 *D–F*	control: *GMRwhite RNAi/UAS-Dcr2 w; GMR-Gal4 UAS-tubulinGFP/* *40D-UAS; tub-Gal80^ts^/+* *shi^ts^*: *GMRwhite RNAi/UAS-Dcr2 w; GMR-Gal4 UAS-tubulinGFP/tub-* *Gal80^ts^; UAS-shi^ts^/+*
Figure 8 *G–I*	control: *w* *ort^1^/ort^5^: w;;ort^1^/ort^5^*
Figure 9	*w/GMRwhite RNAi; Rh4-LexA/LexAop-spGFP1-10 UAS-CD4spGFP11;* *ort-C2-Gal4/*+
Figure 10	*w/GMRwhite RNAi; Rh4-LexA/LexAop-spGFP1-10 UAS-CD4spGFP11;* *ort-C2-Gal4/*+

### Brain preparation

**Figure 1.**
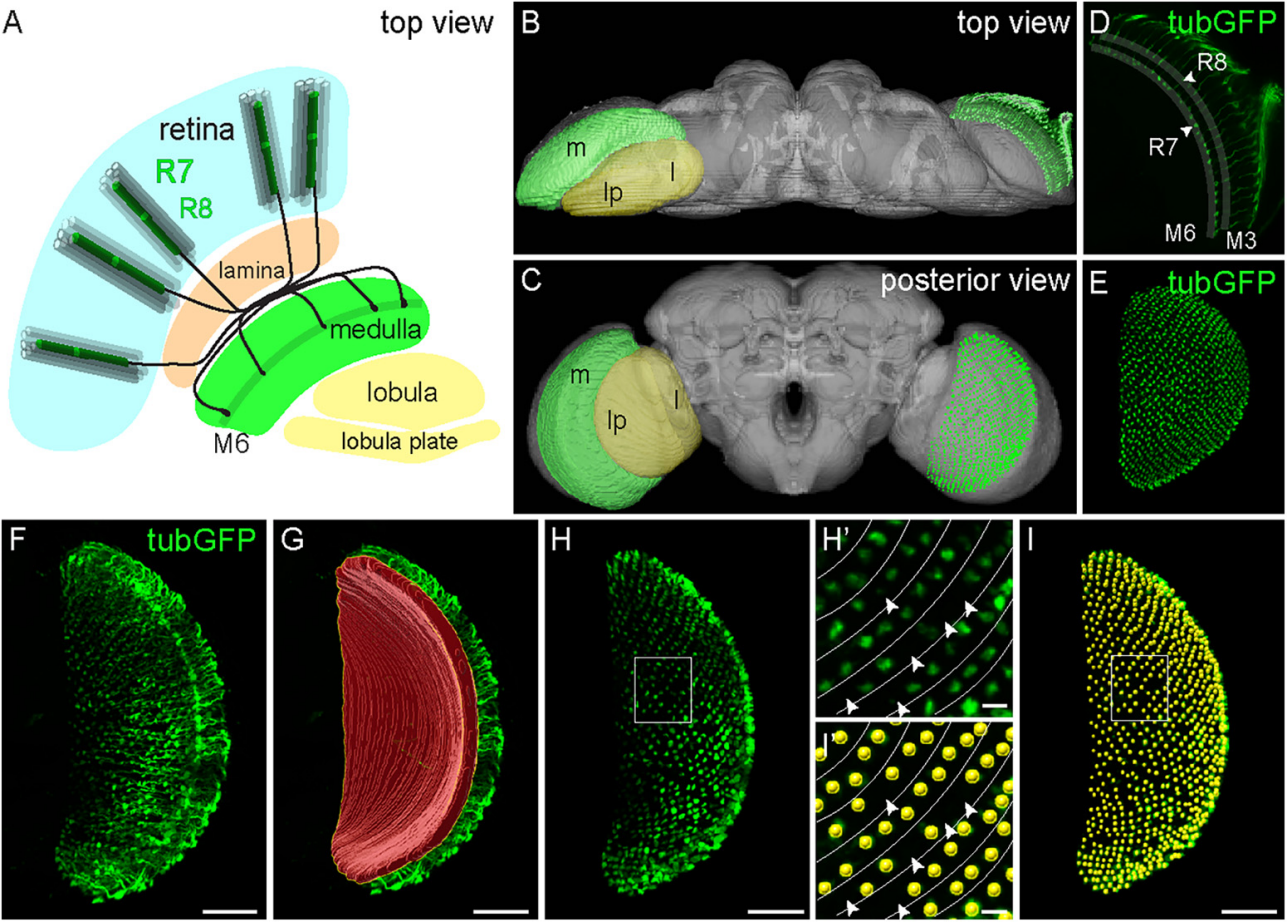
Quantitative analysis of sporadic axonal degeneration in *Drosophila* R7 photoreceptors. ***A***, Scheme of the adult fly visual system. The retinal layer (blue) contains the cell bodies of the PRCs, which are organized in ∼750–800 columnar units (ommatidia) all containing one PRC of each type (R1–R8). R1–R6 (gray columns) project their axons (data not shown) to the lamina (orange). R7 and R8 PRCs (green) project their axons to the medulla (green); R7 termini (black) are found in medulla layer M6, whereas R8 terminates in M3 (data not shown; Fischbach and Dittrich, 1989). ***B***, ***C***, Three-dimensional image stacks of the adult fly brain (gray) depicting the positions of the medulla (green) and the lobula and lobula plate (yellow) in a top (***B***) or posterior (***C***) view (left part of the brain) as well as a *z* projection of R7 axons in a top view (***B***) or the R7 termini in the posterior view (***C***; right part of the brain). ***D***, The *z*-projection of tubulinGFP-labeled R7 and R8 axons imaged in a top brain view. R7 axons terminate in M6 (arrowhead), whereas R8 axons terminate in M3 (arrowhead). ***E***, TubulinGFP-labeled R7 termini in a posterior brain view after extraction from a 3D brain reconstruction. The genotypes in this and all the rest of the figures are included in Table 1. ***F–H***, Identification of R7 axon termini in the M6 medulla layer. Three-dimensional projection of tubulinGFP-labeled R7 and R8 axons in the medulla in a posterior brain view before extraction of R7 termini (***F***), 3D reconstruction of created mask (red surface) to extract R7 termini in M6 (***G***), and R7 termini after extraction (***H***). ***H'***, Close-up of R7 axonal termini in the medulla of flies expressing *UAS-tubulinGFP* driven by *GMR-Gal4*. The organization of medullar R7 termini in rows (lines) facilitates the detection of missing axons upon prolonged light exposure (arrowheads). ***I*, *I'***, A spot is assigned to each R7 axonal terminus (yellow). The total number of dots represents the amount of R7 axon termini in the medulla. Scale bars: ***F–I***, 10 µm; ***H'***, ***I'***, 5 µm.

For medulla imaging, brains were prepared in PBS according to Sugie et al. (2015, 2017) and fixed for 50 min in 4% paraformaldehyde. Brains were stained with mouse anti-Chaoptin (1:25; catalog #24B10, Developmental Studies Hybridoma Bank) and incubated overnight at 4°C in PBS plus 1% Triton X-100 (PBT) containing 0.1% BSA. After washing, secondary antibody (Ab) was incubated for 2 h at room temperature (Alexa 568 conjugated anti-mouse Ab, 1:400; Life Technologies). Brains were then mounted in Vectashield (Vector Laboratories), with the posterior side up, after a second washing step. Insect pins of 0.1 mm diameter (Entomoravia) were used as spacers to keep the brain in its original shape. To visualize the axons in their extension in the medulla (Fig. 1*B*,*D*; see Fig. 10), brains were oriented ventral side up and imaged in a ventral to dorsal orientation (Sugie et al., 2017). Confocal microscopy was performed with an Olympus FV3000 (see Figs. 7–10) or with a Zeiss LSM 780 (Fig. 1; see Figs. 3, 5, 6). Images were processed using Imaris software (Bitplane) and Fiji software (Schindelin et al., 2012).

### Axonal termini counts

To quantify the number of R7 axonal termini in medullas, confocal *z*-stacks of ∼100 µm in depth were acquired with a 40× objective (Zeiss LSM 780) or a 60× objective (Olympus FV3000). The *z*-stack interval was fixed to 1 µm, and the following settings were used: 1 A.U., 1024 frame with LSM 780 or 512 frame with FV 3000, 8 bit with LSM 780 or 16 bit with FV3000. We then reconstructed the stacks in 3D using the Imaris 9.7.2 software. Axonal termini of R7 PRCs project into the M6 medulla layer, which can be masked by using the Imaris surface function. To create a perfect mask, we navigated slide by slide through the 3D-reconstructed medulla in Imaris and manually marked the M6 region containing only R7 axonal termini in every third slide of the reconstructed *z*-stack. We then used the surface function to recreate a compact 3D mask of R7 termini based on the manually drawn regions. In an accompanying study, we developed a software enabling automated mask creation, which was partially used in this study and is described in detail in Nitta et al. (2021). After mask creation, we isolated the signal of the mask using the mask channel function. In this step, we could visualize only the R7 termini by selecting the masked channel in the display adjustment tool. We subsequently aimed to count the number of individual axonal termini by using the spot function. To do so, we added four image processing steps into our protocol. We selected gamma correction values to 1.4, and threshold cutoff values were set up individually for each image, as half of the automatically determined peak of intensity. In addition, we chose the background subtraction function with values suggested by the program, and the Gaussian filter was selected as 0.6 µm. These processing steps allowed us to visualize axonal termini in better quality, and thus facilitate the detection of termini by the spot function.

By using the estimated XY diameter function, we set the size of a terminus to 2 µm (based on healthy axonal size in LD). Additionally, we aimed at avoiding the presence of off-targets and thus used the background subtraction function while keeping the quality thresholds manual. For images obtained with the LSM 780 confocal microscope, the quality threshold was determined to be 2 for tubulinGFP signals and 1 for 24B10-positive axons; for images obtained with the Olympus confocal setup, the quality thresholds were adapted to 300 for 24B10 and 150 for the GFP signal. To provide highly accurate counts and detect early axonal loss, which consists of only 5–10% of the total amount of axonal termini (see Fig. 3*J*,*K*), we used the highly organized structure of medulla and took advantage of its axons organized in lines and rows to verify that the assigned dots belonged to axonal termini (Fig. 1*H'*,*I'*). Off-targets were removed manually from the final count.

### Retinal staining/TUNEL assay

Eyes were removed from heads of *GMRwhite RNAi/w;GMR-Gal4/*+*;UAS-tubulinGFP/*+ female flies in PBS and fixed in 4% paraformaldehyde overnight at 4°C. After washing in 0.1% PBT, eyes were incubated in 50 mg/ml sodium borohydride (Sigma-Aldrich) in PBS for 20 min to remove pigments and subsequently washed in PBS and PBT. The cornea was removed and retinas were stained overnight at 4°C with Phalloidin-iFluor 488 (1:1000; Biomol) in 0.1% PBT BSA. On the next day, retinas were washed with PBT and PBS and embedded directly in Fluoromount (SouthernBiotech) or further processed for TUNEL labeling. TUNEL staining was performed according to manufacturer instructions (In Situ Cell Death Detection Kit TMR red, Roche). For the positive control, retinas were incubated with 300 U/ml DNase I in Tris, pH 7.5, and 1 mg/ml BSA for 10 min at 25°C before TUNEL labeling. Retinas were washed in PBS and mounted with Fluoromount containing DAPI (SouthernBiotech).

### Eye pigment measurements

For brown (ommochrome) eye pigment measurements, we used a protocol adapted from Mackenzie et al. (1999). Fifty heads of 7–10-d-old female flies were homogenized in 150 μl of 2 m HCl and 0.66% sodium metabisulfite (w/v, Sigma-Aldrich). Two hundred microliters of 1-butanol was added, and the mixture was placed on an orbital shaker at 150 rpm for 30 min before being centrifuged at 9000 × *g* for 5 min. The organic layer was removed and washed with 150 μl of 0.66% sodium metabisulfite in dH_2_O and placed back on the orbital shaker for a further 30 min. The organic layer was removed and measured for absorbance at 492 nm. Absorbance was determined with a Nanodrop One C spectrophotometer (Thermo Fischer Scientific). Adult eye pictures were obtained with a Canon EOS 700D camera mounted on a Leica S8AP0 binocular and processed in Fiji (Schindelin et al., 2012).

### Circadian rhythm and sleep behavioral assay

The behavioral trials were conducted using the procedure described previously (Fogg et al., 2014; Yoshii et al., 2015), with some modifications. A DAM5 *Drosophila* activity monitor system (TriKinetics) was used to record locomotor activity in 1 min bins. Individual adult male flies of the genotype *GMRwhite RNAi/Y;GMR-Gal4 UAS-tubulinGFP*/+ from 1 to 4 d old were transferred to recording tubes containing 5% sucrose in 0.9% agar on one end. For LL experiments, male and female flies were entrained to a 12 h LD cycle (light, 4000 lux) at 25°C for 3 d. Subsequently, test flies were subjected to LL conditions for 10 d and control flies to LD cycles for 10 d. Activity recordings were analyzed using ActogramJ software (Schmid et al., 2011). The sleep was defined as previously described (Huber et al., 2004).

### Statistical analysis

Statistical analyses were performed with GraphPad Prism 9.1.0. All quantifications were performed by experimenters who were blind to the genotypes. The distribution of our data was determined using the D'Agostino–Pearson test and the Kolmogorov–Smirnov test (normality test was passed if *p* > 0.05). For data following a Gaussian distribution, we used ordinary one-way ANOVA with Tukey's multiple comparisons between groups. For experiments containing non-normally distributed data, we used the Kruskal–Wallis test and Dunn's multiple comparisons between groups. For the sleep experiment, we used the Mann–Whitney *U* test; *p* values above 0.05 were considered nonsignificant. Statistical details are provided in Table 2.

**Table 2.** Statistics

Figure	Statistical analysis
Figure 2	***B***, One-way ANOVA, *F*_(3.8)_ = 0.24, *p* = 0.8681; *post hoc* comparisons with the first column using Tukey's HSD.
Figure 3	***J***, One-way ANOVA, *F*_(8.146)_ = 255.9, *p* < 0.0001; *post hoc* comparisons with 1dLL using Tukey's HSD are shown on every column. Number of medullas analyzed in brackets, 1dLD (*n* = 18), 1dLL (*n* = 15), 3dLL (*n* = 17), 5dLL (*n* = 10), 7dLL (*n* = 17), 9dLL (*n* = 19), 11dLL (*n* = 19), 13dLL (*n* = 20), 13dLD (*n* = 20). ***K*,** One-way ANOVA, *F*_(8.133)_ = 137.7, *p* < 0.0001; *post hoc* comparisons to 1 dLL using Tukey's HSD are shown on every column. Number of medullas analyzed in brackets, 1dLD (*n* = 21), 1dLL (*n* = 16), 3dLL (*n* = 15), 5dLL (*n* = 11), 7dLL (*n* = 19), 9dLL (*n* = 11), 11dLL (*n* = 14), 13dLL (*n* = 15), 13dLD (*n* = 20).
Figure 4	***A***, One-way ANOVA, *F*_(4.68)_ = 282.3, *p* < 0.0001; *post hoc* comparisons with 13dLD using Tukey's HSD are shown on every column. Number of medullas analyzed in brackets, 13dLD 10,000 lux (*n* = 14), 13dLL 10,000 lux (*n* = 17), 13dLL 4000 lux (*n* = 10), 13dLL 4000 lux males (*n* = 11). ***D***, Mann–Whitney *U* test, *p* < 0.0001. Number of flies used in the experiment, LD (*n* = 28), LL (*n* = 28).
Figure 5	***M***, One-way ANOVA, *F*_(15.184)_ = 92.23, *p* < 0.0001; *post hoc* comparisons using Tukey's HSD are shown between compared conditions; 5dLL (*n* = 13) is compared with 5dLL + 6dDD (*n* = 10) and 5dLL + 6dLD (*n* = 16), 7dLL (*n* = 15) is compared with 7dLL + 4DD (*n* = 21) and 7dLL + 4LD (*n* = 16), 9dLL (*n* = 14) is compared with 9dLL + 2dDD (*n* = 7) and 9dLL + 2dLD (*n* = 10). An additional comparison between 9dLL + 2dDD, 9dLL + 2dLD and 11dLL was added. Control time points shown, 1dLD (*n* = 18), 11dLL (*n* = 12).
Figure 7	***K***, Kruskal–Wallis test, *H* = 149.3, *p* < 0.0001; Dunn's multiple comparisons are shown on every column in comparison with 1dLL control. In addition, comparisons between control and p35 conditions are marked above each line (all nonsignificant). Number of medullas analyzed in brackets: control 1dLL (*n* = 19), control 5dLL (*n* = 20), control 9dLL (*n* = 18), control 13dLL (*n* = 15), control 13dLD (*n* = 23), p35 1dLL (*n* = 20), p35 5dLL (*n* = 21), p35 9dLL (*n* = 20), p35 13dLL (*n* = 20), p35 13dLD (*n* = 18).
Figure 8 *A–C*	Kruskal–Wallis test, *H* = 140.5, *p* < 0.0001; Dunn's multiple comparisons are shown on every *TrpA1* column in comparison with its control. Number of medullas analyzed in brackets, for 13dDD at 20°C control (*n* = 11) and *TrpA1* (*n* = 14); for the control flies at 29°C, 1dDD (*n* = 19), 3dDD (*n* = 20), 5dDD (*n* = 13), 7dDD (*n* = 17), 9dDD (*n* = 17), 11dDD (*n* = 16), 13dDD (*n* = 17); for *TrpA1* flies at 29°C, 1dDD (*n* = 23), 3dDD (*n* = 18), 5dDD (*n* = 18), 7dDD (*n* = 15), 9dDD (*n* = 16), 11dDD (*n* = 14), 13dDD (*n* = 15).
Figure 8 *D–F*	Kruskal–Wallis test, H = 61.98, *p* < 0.0001; Dunn's multiple comparisons are shown between *shi^ts^* and its corresponding control. Number of medullas analyzed in brackets: control 1dLL (*n* = 18), control 9dLL (*n* = 16), control 13dLL (*n* = 12), *shi^ts^* 1dLL (*n* = 15), *shi^ts^* 9dLL (*n* = 15), *shi^ts^* 13dLL (*n* = 12).
Figure *8G–I*	One-way ANOVA, *F*_(11.180)_ = 349.7, *p* < 0.0001; *post hoc* comparisons were performed using Tukey's HSD. Significance levels between *ort^1^/ort^5^* and its equivalent control (*w*) upon various light exposure times are depicted on the graph. Number of medullas analyzed in brackets, *w* 1dLD (*n* = 19), *ort^1^/ort^5^* 1dLD (*n* = 17), *w* 1dLL (*n* = 15), *ort^1^/ort^5^* 1dLL (*n* = 16), *w* 3dLL (*n* = 18), *ort^1^/ort^5^* 3dLL (*n* = 18), *w* 5dLL (*n* = 12), *ort^1^/ort^5^* 5dLL (*n* = 11), *w* 7dLL (*n* = 17), *ort^1^/ort^5^* 7dLL (*n* = 16), *w* 7dLD (*n* = 17), *ort^1^/ort^5^* 7dLD (*n* = 16).
Figure 9	***J*,** Kruskal–Wallis test, *H* = 94.29, *p* < 0.0001; Dunn's multiple comparisons are shown on every column in comparison with 1dLL. Number of medullas analyzed in brackets, 1dLD (*n* = 16), 1dLL (*n* = 16), 3dLL (*n* = 15), 5dLL (*n* = 20), 7dLL (*n* = 17), 9dLL (*n* = 15), 11dLL (*n* = 12), 13dLL (*n* = 12), 13dLD (*n* = 27). ***K***, Kruskal–Wallis test, *H* = 96.78, *p* < 0.0001; Dunn's multiple comparisons are shown on every column in comparison with 1dLL.

## Results

### Prolonged light exposure induces progressive axonal degeneration in photoreceptors

As a first step to establish a reliable setup for the induction of sporadic axonal degeneration in the nervous system, we studied the impact of prolonged light exposure on the visual system of adult flies. The *Drosophila* visual system consists of ∼750–800 independent unit eyes called ommatidia, which are organized in a crystalline-like arrangement (Tomlinson and Ready, 1987; Wolff and Ready, 1991). Each ommatidium is composed of eight PRCs that detect light and project retinotopically into the optic lobe, where visual processing occurs. Photoreceptor R1–R6 project axons to the first neuropil of the optic lobe, the lamina, whereas R7 and R8 contact their postsynaptic partners in the second neuropil, the medulla (Fischbach and Dittrich, 1989; Takemura et al., 2008). Although cell death in the fly retina has been used extensively in neurodegeneration studies (Lenz et al., 2013), here we chose to monitor PRC axonal termini to concentrate on axonal degeneration. In particular, we focused on R7 PRCs because their axon termini all reach a precise medulla layer (M6), where they display a highly ordered distribution and constant numbers (∼750–800; Fig. 1*A*; Fischbach and Dittrich, 1989; Takemura et al., 2008). R7 axons and axonal termini can be readily visualized with antibodies against chaoptin (24B10; Fujita et al., 1982) or by labeling PRCs genetically with *GMR-Gal4* driving expression of *UAS-tubulinGFP* (Fig. 1*B–E*). Expression of *tubulinGFP* was detected along both R7 and R8 axons when imaging the medulla in a dorsal to ventral orientation (Fig. 1*B*,*D*; Sugie et al., 2015). To obtain reliable counts of R7 axon terminals only, we first generated *z*-scans of adult optic lobes, including the entire medulla, by imaging them in a posterior to anterior orientation (Fig. 1*C*). We then reconstructed all labeled axons in 3D and observed the R7 photoreceptor axons until they terminate in the M6 medulla layer (Fig. 1*F*). We then marked the R7 termini in M6 layer of the 3D reconstructed medullas to generate a 3D mask (Fig. 1*G*), which we used to isolate the axon termini layer (Fig. 1*H*). Each axon terminus was identified with a dot (see above, Materials and Methods), and the total number of dots was quantified (Fig. 1I). By imaging the medulla in this particular orientation and having extracted the M6 medulla region, we were able to reconstruct a complete array of R7 axonal termini, preserving their highly organized distribution. This allowed us to identify each missing axon (Fig. 1*H'*,*I'*), particularly at the very early stages of neurodegeneration, in which only few axons are lost (see Fig. 3). Thus, this technique allows for a precise and reliable counting of axonal termini of R7 in adult brains.

**Figure 2.**
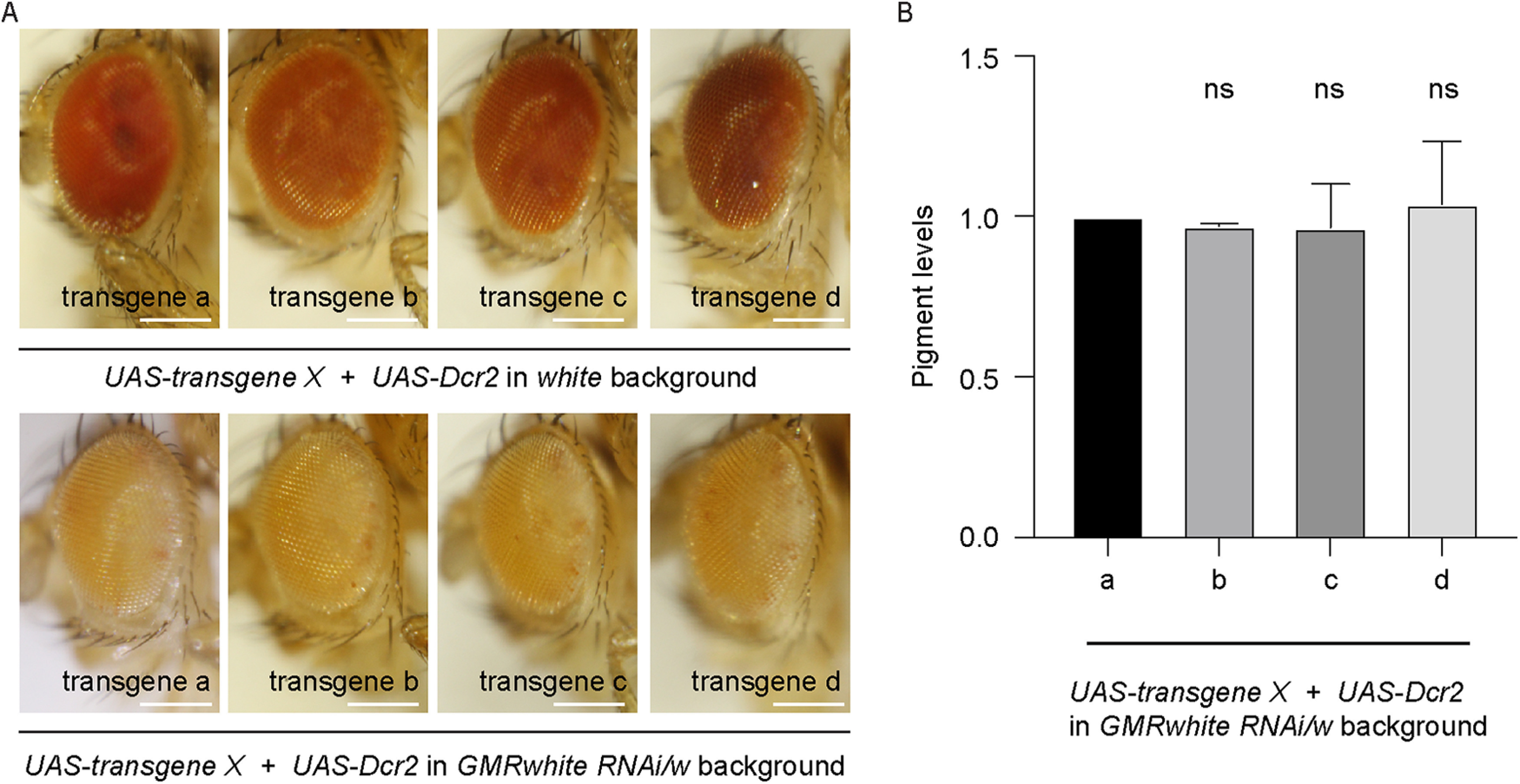
The establishment of a fly line in which eye pigment levels are independent of the included transgenes. Eye color is homogeneous in a background containing *GMRwhite RNAi/w* and *UAS-Dcr2* independently of the addition of different *w*^+^-expressing transgenes. ***A***, Top, The eyes of *white* with *UAS-transgene X* in the presence of *UAS-Dcr2*. Bottom, The eyes of *GMRwhite RNAi/w* flies with *UAS-transgene X* and *UAS-Dcr2*. Scale bar, 250 µm. The transgenes used are listed in Table 1. ***B***, Brown pigment content in eyes of 7–10-d-old *GMRwhite RNAi/w* female flies with *UAS-transgene X* and *UAS-Dcr2*. Bars represent mean ± SD of three experiments (ns, not significant). Pigment levels in the *GMRwhite RNAi/w;GMR-Gal4/UAS-transgene a;UAS-tubulinGFP/UAS-Dcr2* flies were arbitrary, set to 1.0. Statistical details for this and all graphs in the following figures are included in Table 2.

We exposed flies to a light intensity of ∼10,000 lux corresponding to light measured in the shade of a summer day (Schlichting et al., 2019). Photoreceptors implement multiple protective mechanisms to keep their activation level and their output to downstream neurons within a working range (Stavenga, 1995; Juusola and Hardie, 2001; Sugie et al., 2015, 2018; Bai and Suzuki, 2020). We found that R7 axons are highly resilient in wild-type flies as R7 axon loss could be observed only starting after 3 weeks of continuous ∼10,000 lux light exposure (data not shown). This is primarily because of the fact that optical isolation of retinal ommatidia by the pigment cells protects retinal photoreceptors from excessive exposure to light (Shoup, 1966; Schraermeyer and Dohms, 1993; Lee and Montell, 2004; Bulgakova et al., 2010; Ferreiro et al., 2017). To thus facilitate the initiation of degeneration, we decreased pigment content in the retina by modulating the expression of the *white* gene (O'Hare et al., 1984; Pepling and Mount, 1990; Mackenzie et al., 2000). In *white* mutant flies, degeneration started quickly and was widespread (see Fig. 8*G–I*; data not shown). To generate instead a situation in which axon degeneration is sporadic and progressive, we took advantage of a *GMRwhite RNAi* transgene to knock down less efficiently *white* expression in the eye, thus yielding a yellow eye color (Fig. 2*A*; Lee and Carthew, 2003). In combination with *UAS-Dcr2*, this genotype displayed a homogenous eye color, independently of the inclusion of additional *w*^+^ transgenes (Fig. 2*A*,*B*).

**Figure 3.**
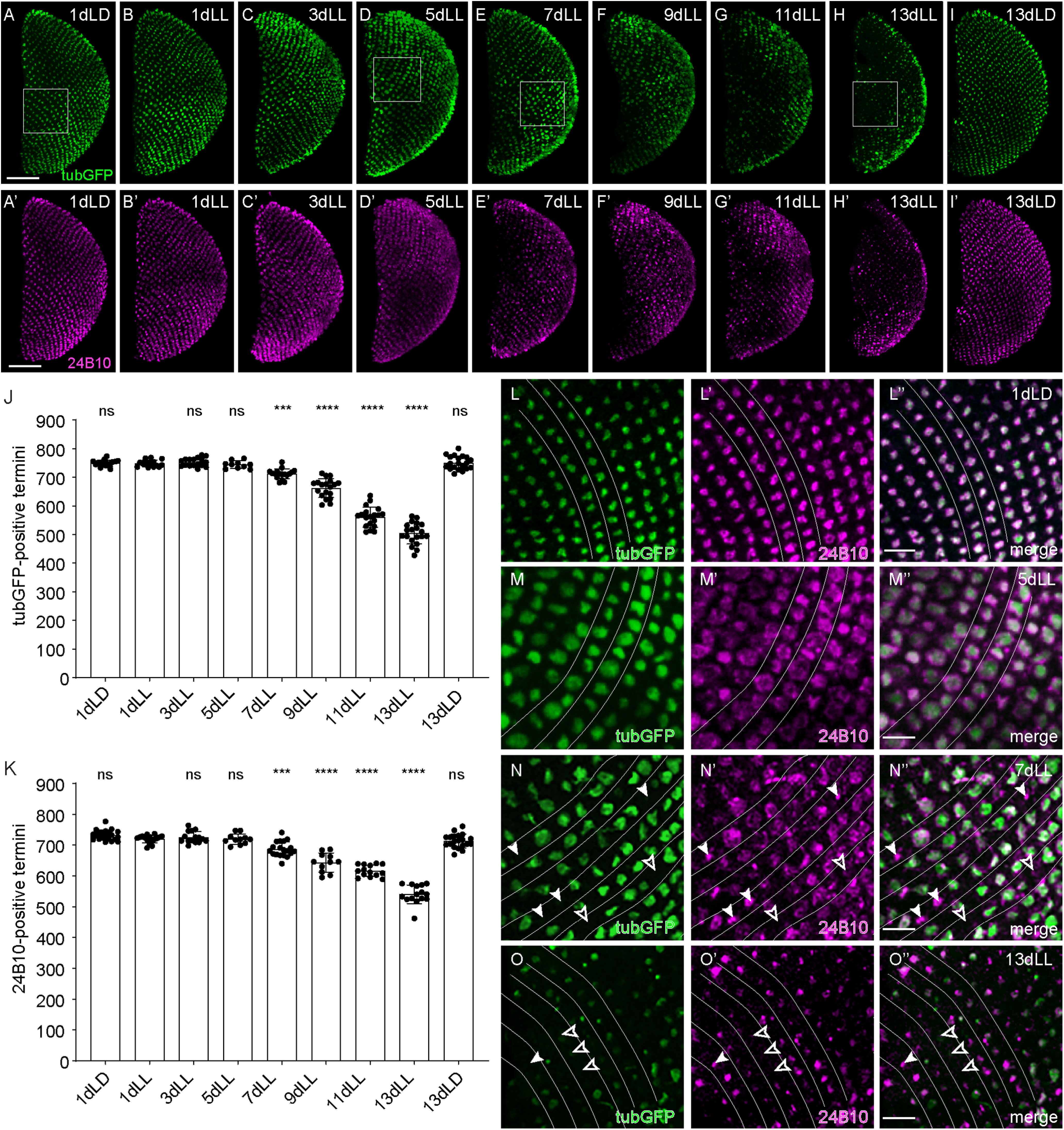
Light-dependent progressive degeneration of photoreceptor axons in the *Drosophila* medulla. ***A–I***, ***A'–I'***, Axonal termini of R7 in the medulla of flies exposed to LL for various days or to control conditions for 1 d (***A***, ***A'***) or 13 d (***I***, ***I'***) in an LD cycle. *UAS-tubulinGFP* driven by *GMR-Gal4* (green; ***A–I***). Chaoptin (24B10; magenta; ***A'–I'***). Squares depict the position of the close-ups in ***L–O***. Scale bar, 50 µm. ***J***, TubulinGFP-positive R7 termini counts in the medulla of flies exposed for various time to LL or control conditions (LD). ***K***, The 24B10-positive R7 termini counts in the medulla of flies exposed for various time to LL or control conditions (LD). Error bars indicate mean ± SD (ns, not significant, *** *P* < 0.001, **** *P* < 0.0001). ***L–O***, Close-ups of R7 axonal termini in the medulla of flies at 1dLD (***L–L”***), 5dLL (***M–M”***), 7dLL (***N–N”***), and 13dLL (***O–O”***) labeled with *UAS-tubulinGFP* driven by *GMR-Gal4* (green; ***L–O***), chaoptin (24B10; magenta; ***L'–O'***), or merged (***L”–O”***). Filled arrowheads point to degenerating termini that have lost tubulinGFP expression but still retain the membrane marker 24B10. Empty arrowheads point at missing termini. Lines are drawn between rows of axonal termini. Scale bar, 10 µm.

In these flies, a small subset of R7 axonal termini started disappearing after 7 d of LL exposure (Fig. 3*E*,*J*,*N*). Axons degenerated progressively between 7 and 13 d of light exposure (Fig. 3*J*,*F*,*G*). By 13 d of light exposure, only ∼60% of the 750–800 axonal termini found 1 d after eclosion (Fig. 3*A*,*J*) were still present (Fig. 3*J*,*H*,*O*) and axon counts dropped further to ∼400 termini after 22 d of light exposure (380.20 ± 38.33, *n* = 15). Axon loss occurred randomly throughout the neuropil, without obvious signs of regionalization.

The numbers of axonal termini were unaffected by a cyclic exposure of 12 h of light at 10,000 lux and 12 h of darkness for 13 d (Fig. 3*I*,*J*). To distinguish between loss of tubulin and loss of axons, we immunolabelled the medulla with antibodies against chaoptin (24B10), which decorates the membrane of R7 and R8 axons in the medulla but only R7 in the M6 layer. The 24B10-positive axon numbers progressively diminished upon LL exposure from days 7–13 in a way that mimics the loss of tubulin (Fig. 3*K*,*E'*–*H'*,*N'*,*O'*). They were unaffected by LD exposure (Fig. 3*K*,*I'*). Close-up optical sections at the axonal termini showed that after the first days in constant light, axonal termini first swelled (compare Fig. 3*M*,*M'* with Fig. 3*L*,*L'*) before they started degenerating (Fig. 3*N*,*N'*). Because R7 axonal termini are organized in parallel rows, axon degeneration can be precisely and reliably monitored in this system (Fig. 3*L–O”*). After 7 d in constant light, axon degeneration became apparent, with individual termini missing (Fig. 3*N–N”*). At 13dLL, axonal degeneration was widespread in the medullas, and we observed both degenerating termini in which only chaoptin labeling was left and a large number of missing termini (Fig. 3*O–O”*).

**Figure 4.**
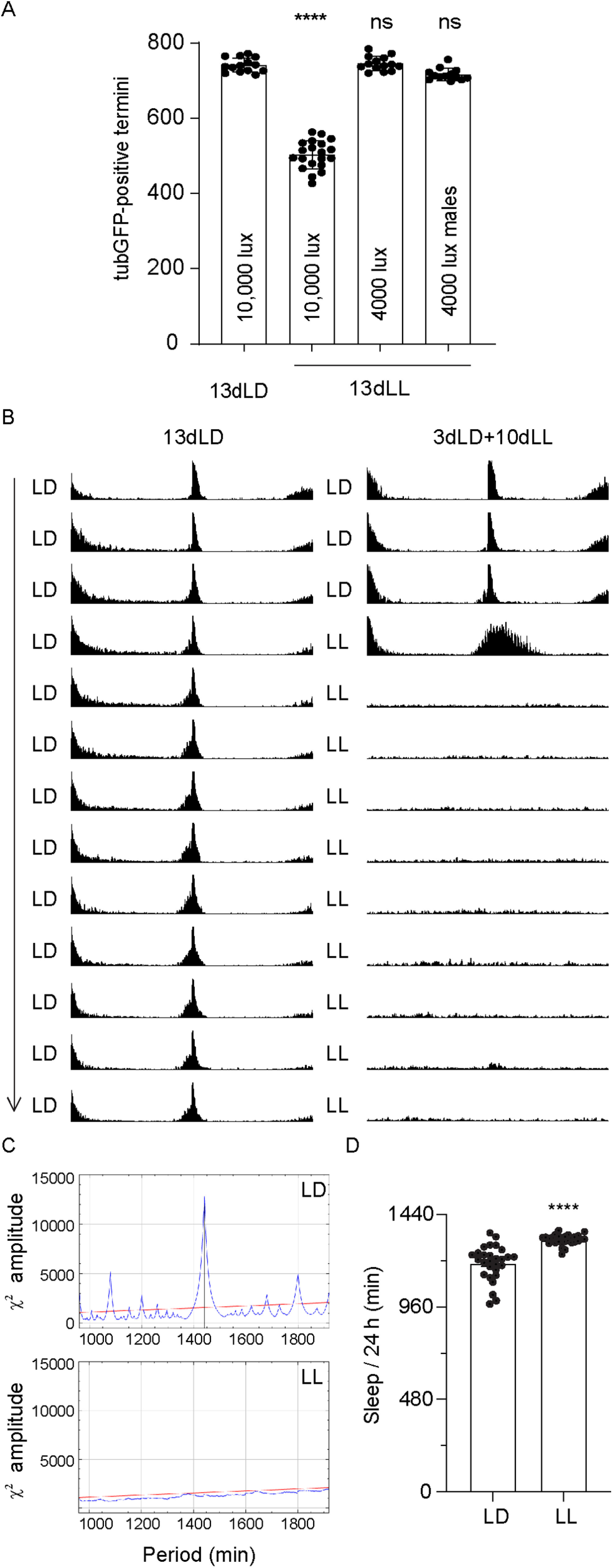
Disruption of circadian rhythm does not cause axonal degeneration. ***A***, TubulinGFP-positive R7 termini in the medulla of *GMRwhite RNAi/w;GMR-Gal4/*+*;UAS-tubulinGFP/*+ flies exposed to 13 d LD or 13 d LL of 10,000 or 4000 lux, or males *GMRwhite RNAi/Y;GMR-Gal4 UAS-tubulinGFP/*+ exposed to 13 d in constant light of 4000 lux. ***B***, Actogram showing the average locomotor activity of *GMRwhite RNAi/Y;GMR-Gal UAS-tubulinGFP*/+ flies in LD (left) and LL (right) at 4000 lux. Flies were entrained to an LD cycle at 25°C for 3 d (top 3 actograms). Subsequently, test flies were subjected to LL conditions for 10 d (right) and control flies to LD cycles for 10 d (left). ***C***, The χ^2^ periodogram records of LD (top) or LL (bottom) conditions in *GMRwhite RNAi/Y;GMR-Gal4 UAS-tubulinGFP*/+ flies. The blue line shows a period of 1440 min (24 h) for flies in LD, whereas no periodicity was detected in LL flies. The red line indicates a significant level of *p* = 0.05. ***D***, Total amount of sleep of *GMRwhite RNAi/Y;GMR-Gal4 UAS-tubulinGFP*/+ flies exposed to LD and LL at an intensity of 4000 lux during 24 h. Sleep was defined as an absence of movement for >5 min. Error bars indicate mean ± SD (ns, not significant, **** *P* < 0.001).

Because constant light exposure abolishes circadian rhythm in the fruit fly (Konopka et al., 1989), we asked whether the light-dependent axonal degeneration observed in the medullas was because of a disruption of circadian rhythm. For this, we exposed flies to a weaker light intensity, which does not induce degeneration of R7 axons (4000 lux, Fig. 4*A*), and monitored their activity over a 10 d period either in LL (at 4000 lux) or LD (12 h of light at 4000 lux and 12 h of darkness). As expected, flies in LL lost their rhythm after 1 d of constant mild light exposure (Fig. 4*B*,*C*), showing that a mere disruption of cyclicity does not per se induce neurodegeneration in the medulla. In addition, we observed that this condition induced a slight increase of total sleep time in the LL flies rather than sleep loss (Fig. 4*D*).

Together, we developed a model of sporadic and progressive axonal degeneration in the medulla, in which it is possible to quantitatively evaluate initial phases of axon loss.

### Reversibility of light-dependent axonal damage in R7 photoreceptors

**Figure 5.**
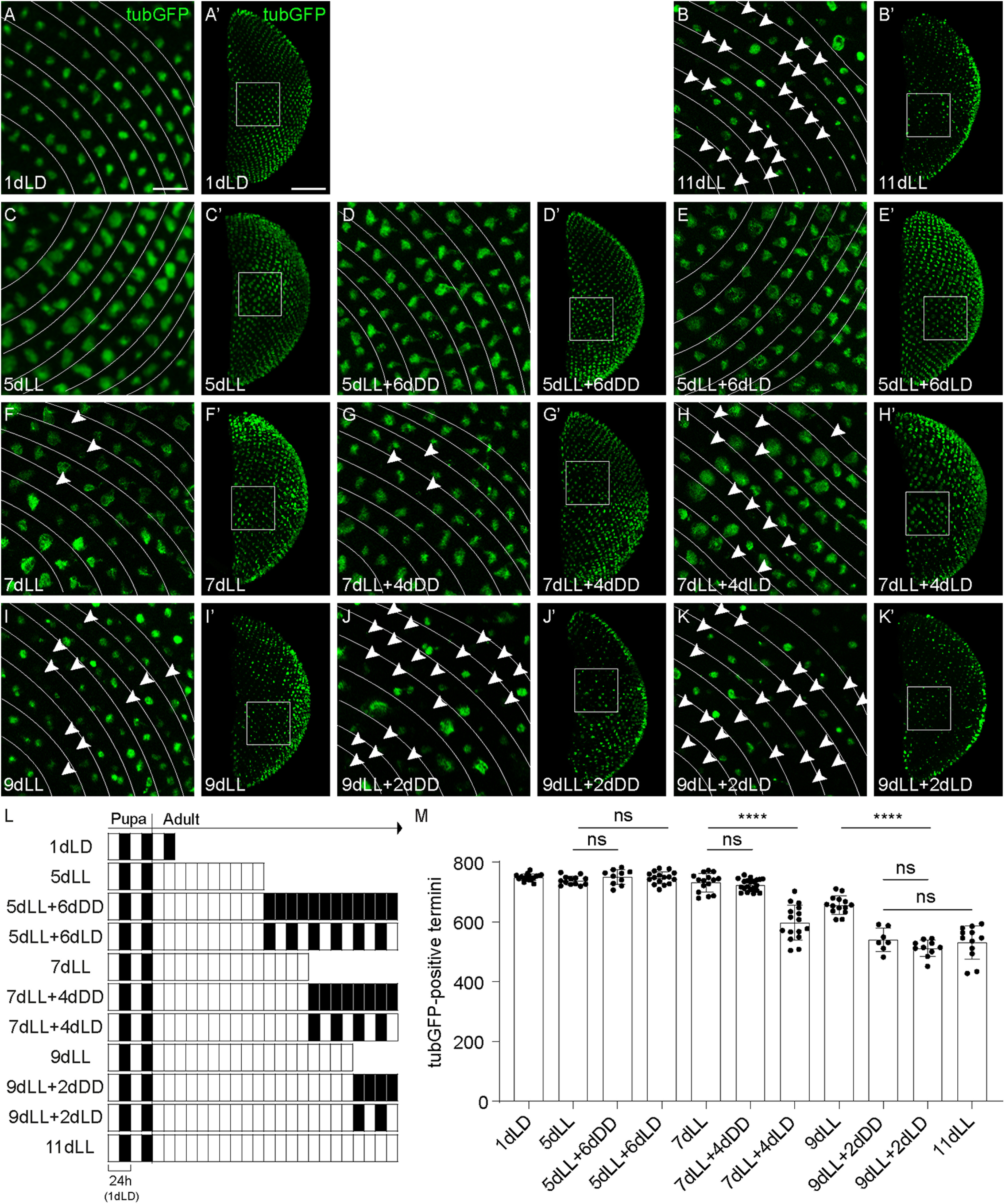
Reversibility of light-induced axonal damage in R7 photoreceptors. ***A–K***, Close-ups of medullas showing axonal termini of R7 in flies expressing *UAS-tubulinGFP* (green) driven by *GMR-Gal4* exposed to various light and dark cycles (***L***). Arrowheads point at missing termini. Lines are drawn between rows of axonal termini. Scale bar, ***A–K***, 10 µm. ***A'–K'***, Corresponding full medullas are depicted. Squares highlight regions of individual close-ups. Scale bar, ***A'–K'***, 50 µm. We considered 1dLD as a nondegenerated condition (***A***, ***A'***), whereas 11dLL was chosen as a condition of strong axonal loss (***B***, ***B'***). ***L***, Scheme of the various illumination conditions used in this experiment. Development proceeded in LD. At eclosion, adult flies were exposed to LL for various time periods (white). A fraction of flies was put back to DD (black) or in an LD cycle (black/white). ***M***, TubulinGFP-positive R7 termini numbers in the medulla of flies exposed to various light and dark cycles. Error bars indicate mean ± SD (ns, not significant, **** *P* < 0.0001).

We sought to identify the time point at which axonal degeneration was initiated. Five days of constant light exposure (5dLL) did not lead to R7 axonal loss in the medulla, but R7 axons underwent morphologic changes, becoming enlarged (Fig. 3*D*–*D'*,*J*, *K*, *M–M”*). We wondered whether axons at 5dLL are already damaged and whether a fraction of them is potentially committed to initiate degenerative processes. We thus placed those flies back into the dark (5dLL + 6dDD) or exposed them to a light/dark cycle (5dLL + 6dLD) and examined them at day 11 (Fig. 5*L*,*C–E'*,*M*). Counts of tubulinGFP-positive termini in the medullas of 5dLL, 5dLL + 6dDD, or 5dLL + 6dLD flies were not distinguishable from those of control animals (Fig. 5*M*). This is in strong contrast to the widespread axonal degeneration observed in 11dLL flies (Fig. 5*B*,*B'*,*M*). Thus, the light-induced changes that had taken place in the R7 axons at 5dLL are reversible, and the axons are not yet terminally committed to degenerate. Interestingly, the swelling presented by the axon termini in flies exposed to 5dLL (Fig. 5*C*,*C'*) was reversed in the 5dLL + 6dDD condition, implying that axon swelling is a reversible modification at this stage (Fig. 5*D*,*D'*). Axon swelling, however, was still present in the medulla of 5dLL + 6dLD, indicating that prolonged D phases are needed for the swelling to disappear (Fig. 5*E*,*E'*).

At day 7 of continuous light exposure (7dLL), first individual axons started to lose the sensitive tubulinGFP signal (Fig. 5*F*,*F'*,*M*). This situation was not worsened 4 d later if the flies were kept in the dark (7dLL+ 4dDD), suggesting that no additional axons had been terminally committed to degenerate at this stage (Fig. 5*G*,*G'*,*M*). Axon swelling of remaining axons was also reduced in this condition. Nonetheless, if the 7dLL flies were placed in a light/dark cycle, progressive axonal degeneration became apparent (Fig. 5*H*,*H'*,*M*). The final outcome was intermediate between that of 9dLL exposure and 11dLL exposure (Fig. 5*M*). This observation suggests that at 7dLL some axons have accumulated reversible damage and that further light exposure is necessary to drive these axons into degeneration.

After 9 d of continuous light exposure (9dLL), the medulla was obviously damaged, with many missing axons (Fig. 5*I*,*I'*,*M*). Putting the flies back in the dark for 2 d (9dLL+ 2dDD) failed to slow down the progressive axonal degeneration, and medullas of these flies were undistinguishable from those of flies exposed to 11dLL (Fig. 5*J*,*J'*,*B*,*B'*,*M*) or to those of flies exposed for 9dLL+ 2dLD (Fig. 5*K*,*K'*,*M*). Thus, at day 9 of light exposure, axon damage may be irreversible, and a fraction of axons might be already committed to degeneration.

Together, these data suggest that R7 axons slowly accumulate reversible damage as a result of constant activation. Axons individually reach a level at which the accumulated damage initiates degeneration, a point of no return.

### Apoptosis is not associated with axonal degeneration

**Figure 6.**
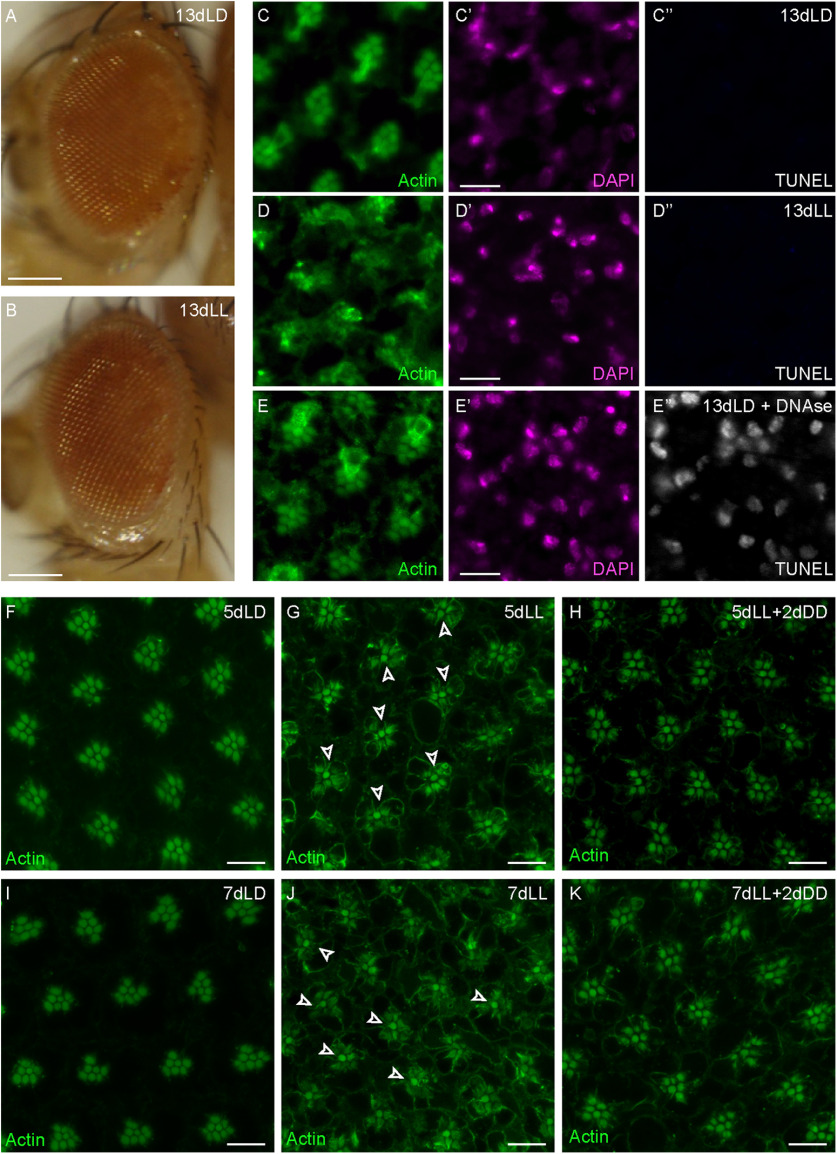
Apoptosis is not associated with axonal degeneration. ***A***, ***B***, Eyes of flies kept for 13 d in an LD cycle (***A***) or for 13 d in LL (***B***). Scale bar, 250 µm. ***C–E'***, Optical cross-sections through the retina of adult flies exposed to 13 d of either control (13dLD in ***C–C''***) or constant light conditions (13dLL in ***D–D”***). Eyes were stained with FITC-Phalloidin to highlight the actin-enriched rhabdomeres (green) and DAPI to mark the nuclei (magenta). TUNEL labeling (white) showed no signal except in the positive control, in which 13dLD eyes (***E–E”***) were submitted to a DNase treatment before TUNEL detection. Scale bar, 10 µm. ***F–K***, Optical cross-sections through the retina of *GMRwhite RNAi/w;GMR-Gal4/*+*;UAS-tubulinGFP/*+ adult flies exposed to control (LD) or LL conditions for 5 (***F***, ***G***) or 7 d (***I***, ***J***) or exposed to 5 or 7 d of constant light followed by 2 d of constant darkness (***H***, ***K***). Eyes were stained with FITC-Phalloidin to highlight the actin-enriched rhabdomeres (green). Arrowheads point to R7 (***G***, ***J***). Scale bar, 10 µm.

Axonal degeneration is an early event in NDs and precedes cell death (Cavanagh, 1979; Adalbert and Coleman, 2013; Neukomm and Freeman, 2014; Tagliaferro and Burke, 2016). To monitor whether axonal degeneration of R7 photoreceptor cells was a consequence of cell death or was preceding it in our model system, we first monitored the cornea. Eyes become rough in conditions in which individual PRCs are missing or when the number, arrangement, or identity of PRCs is modified (Tomlinson et al., 1987, 1988; Basler et al., 1991; Van Vactor et al., 1991). Flies exposed to 13 d of constant light (13dLL) did not show any detectable defects in corneal morphology (Fig. 6*B*), indicating that widespread cell death was not taking place at this stage. To address specifically whether individual PRCs might be undergoing apoptosis, we exposed flies to LD or LL treatments for various periods of time and performed TUNEL staining to detect apoptotic nuclei within the retinas (Fig. 6*C–E”*). Even after 13 d in constant light, we did not detect TUNEL-positive PRC nuclei (Fig. 6*D”*), suggesting that apoptotic cell death had not started at a time point at which axonal degeneration was at full pace (Fig. 3*H*,*H'*,*J*,*K*; Nitta et al., 2021). Nuclear morphology was similar between controls and light-exposed flies (Fig. 6*C'*,*D'*). We observed a reported remodeling of the actin-rich rhabdomeres starting after 5 d of light exposure (Fig. 6*G*). Interestingly, R7 rhabdomeres seemed to be less affected than those of R1–R6 by this light-dependent actin remodeling after 5 or 7 d in constant light (Fig. 6*G*,*J*). This type of actin redistribution in photoreceptors was already observed after short-term light exposure and shown to be reversible in darkness (Kosloff et al., 2003). We thus tested whether we could reverse this phenotype by putting light-exposed flies (5dLL or 7dLL) back in darkness. A large majority of rhabdomeres recovered after 2 d in complete darkness (Fig. 6*H*,*K*), thus suggesting that these changes at the rhabdomeres are not indicative of a permanent cell damage.

**Figure 7.**
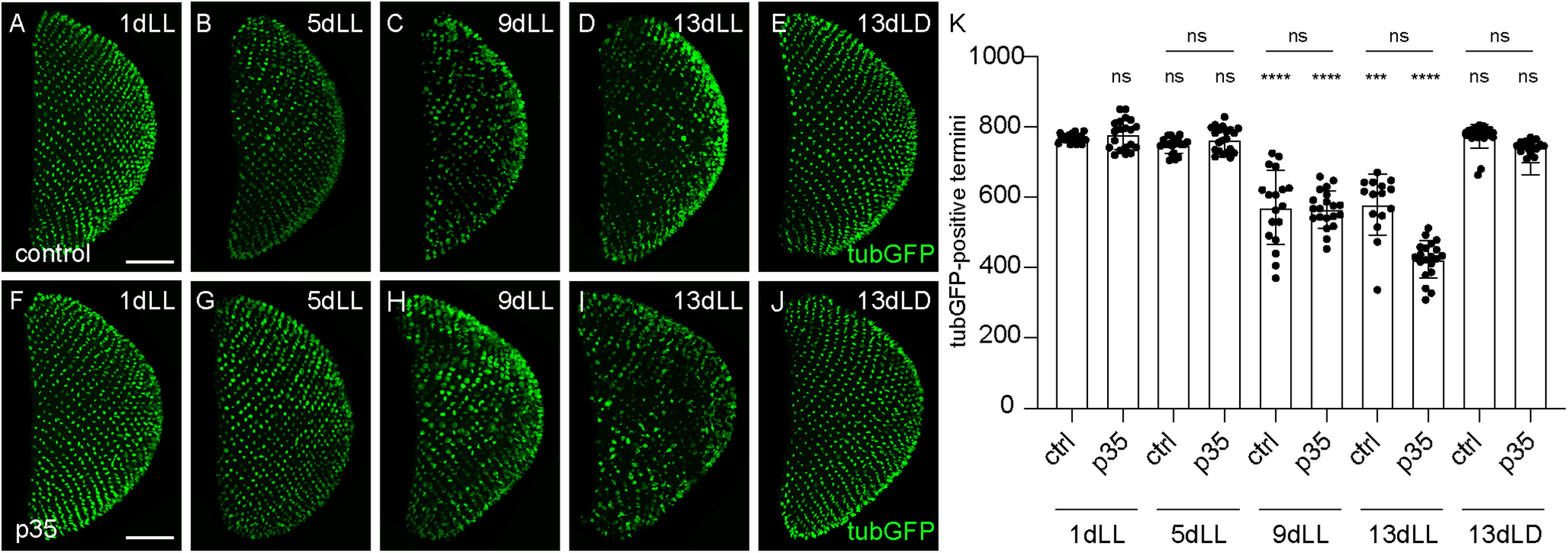
Expression of the caspase inhibitor p35 does not suppress axonal degeneration. ***A–J***, Axonal termini of R7 in the medulla of control (***A–E***) or of flies expressing p35 (***F–J***) exposed for 1, 5, 9, and 13 d to LL or to control 13dLD. R7 axonal termini are revealed by *UAS-tubulinGFP* expression (green). Scale bar, 50 µm. ***K***, TubulinGFP-positive R7 termini counts in the medulla of flies expressing p35 or of control flies exposed for various times to LL or to control 13dLD. Error bars indicate mean ± SD (ns, not significant, *** *P* < 0.001, **** *P* < 0.0001).

To independently validate that apoptosis was not involved in light-induced axonal degeneration during the time frame of our experiments, we expressed in PRCs the baculovirus caspase inhibitor p35 that suppresses apoptosis and analyzed axonal termini numbers after 1, 5, 9, and 13 d in LL (Fig. 7*A–D*, *F–I*; Zhou et al., 1997). Expression of p35 did not modify termini numbers after 13 d in a light/dark control condition (Fig. 7*J*,*K*). Importantly, it did not rescue axonal degeneration at 9 or 13 d in constant light (Fig. 7*H*,*I*,*K*), indicating that apoptosis is not associated to the initial steps of axonal degeneration.

### Neurotransmission is involved in axonal degeneration

**Figure 8.**
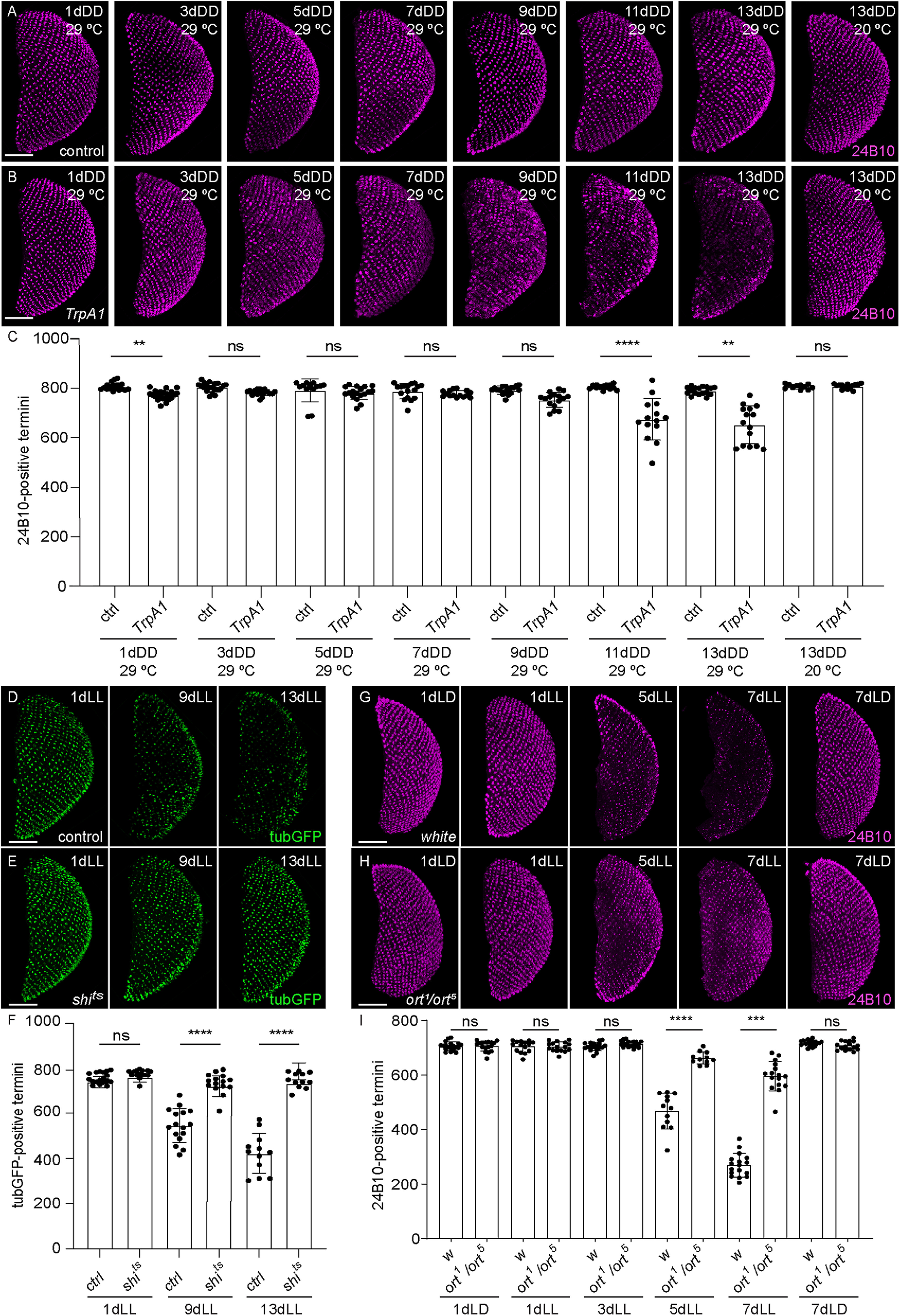
Neurotransmission is involved in the axonal degeneration process. ***A***, ***B***, Axonal termini of R7 in the medulla of flies expressing *UAS-TrpA1* or control flies kept in DD for various periods of time, either at 29°C (test condition) or at 20°C (control). Magenta represents termini stained with 24B10. Scale bar, 50 µm. ***C***, Graph of 24B10-positive R7 termini counts in the medulla of *UAS-TrpA1*-expressing or control flies kept in DD for various periods of time, either at 29°C (test condition) or at 20°C (control). ***D***, ***E***, Axonal termini of R7 in the medulla of flies expressing *UAS-shi^ts^* in (***E***) and control flies in (***D***) exposed for 1, 9, or 13 d to LL. Green represents termini expressing *UAS-tubulinGFP* driven by *GMR-Gal4*. Scale bar, 50 µm. ***F***, Graph of tubulinGFP-positive R7 termini counts in the medulla of flies expressing *UAS-shi^ts^* or control flies exposed for 1, 9, or 13 d to LL. ***G***, ***H***, Axonal termini of R7 in the medulla of *white* flies in (***G***) or of *ort* mutant flies in (***H***) exposed for various times to LL or to control LD conditions for 1 or 7 d. Magenta represents termini stained with 24B10. Scale bar, 50 µm. ***I***, Graph of 24B10-positive R7 termini counts in the medulla of control *white* flies or *ort* mutant flies exposed for 1, 3, 5, or 7 d to LL or kept for 1 or 7 d in LD. Error bars indicate mean ± SD (ns, not significant, ** *P* < 0.01, ^,***^ *P* < 0.001, ^,****^ *P* < 0.0001).

Recent evidence indicates that synaptic failure in AD is at least partly induced by neuronal hyperactivity in the early stages of the disease, and this mechanism could also be involved in developing a late-onset autosomal dominant inherited form of PD (Nuriel et al., 2017; Hector and Brouillette, 2020; Lucumi Moreno et al., 2021). We thus tested whether activity of PRCs is linked to the axonal degeneration described in our model. First, we tested whether we could induce neurodegeneration in complete darkness by acutely activating R7 using the heat-sensitive *Drosophila* TrpA1 channel (Hamada et al., 2008; Pulver et al., 2009). Indeed, activation of photoreceptors by TrpA1 at 29°C was sufficient to induce axonal degeneration in the dark (Fig. 8*B*), starting at 11 d after shifting to the restrictive temperature (Fig. 8*C*). Although TrpA1 might activate R7 at nonphysiological levels compared with light, these data indicate that activation of PRCs can induce axonal degeneration. As a next step, we expressed in R7 the temperature-sensitive transgene *UAS-shibire*^ts^ (*UAS-shi*^ts^), which prevents endocytosis at temperatures exceeding 29°C, thus rapidly stopping synaptic vesicle recycling and neurotransmitter release (Kitamoto, 2001). To avoid developmental defects caused by the leaky expression of *UAS-shi^ts^*, we included a *tubGal80^ts^* transgene in the experimental genotype (McGuire et al., 2003). Flies thus developed at the permissive temperature and were shifted to the restrictive temperature at eclosion, allowing for *UAS-shi^ts^* expression and dominant negative activity only at adult stages (see above, Materials and Methods). In contrast to the degeneration observed in control flies (Fig. 8*D*,*F*), flies expressing *UAS-shi^ts^* in their PRCs and shifted to the restrictive temperature did not show axonal degeneration during prolonged light exposure, even after 13 d of constant light exposure (Fig. 8*E*,*F*).

Because *shi^ts^* impairs endocytic function, we sought to pinpoint more specifically the involvement of neurotransmission and to rule out potential additional effects of hindering endocytosis by Shi^ts^. We thus blocked the postsynaptic response to PRC activation, leaving PRC activity intact. Fly photoreceptors are inhibitory and release histamine, which activates the Ort histamine chloride channel promoting hyperpolarization of R7 postsynaptic neurons in the medulla (Gisselmann et al., 2002; Gao et al., 2008; Pantazis et al., 2008; Liu and Wilson, 2013; Schnaitmann et al., 2018). However, we did not succeed in establishing a line containing an *ort* mutant (Gengs et al., 2002) in the *GMRwhite RNAi* background. As an alternative, we therefore examined a transallelic *ort^1^/ort^5^* combination in *white* mutant flies. Exposure of adult *white* flies to constant light gave rise to a very strong degeneration after only 5 d of exposure, as detected by 24B10 staining (Fig. 8*G*,*I*). With this experiment, we thus confirmed previous results obtained in the retina, showing that pigments protect photoreceptor cell bodies from excessive exposure to light (Lee and Montell, 2004; Bulgakova et al., 2010; Ferreiro et al., 2017). The presence of *ort^1^/ort^5^* protected flies against axonal degeneration, in fact decreasing the loss of axons after 5 and 7 d of constant light exposure (Fig. 8*H*,*I*). A similar protection was obtained in homozygous *ort^5^* mutant flies (24B10-positive termini at 5dLL in *ort^5^* 626.00 ± 47.58, *n* = 19 vs *white* 282.40 ± 68.91, *n* = 19, unpaired *t* test with Welch's correction, ****p < 0.0001). It is conceivable that in the *white* mutant background the mechanisms of degeneration might involve additional components than those acting in pigmented eyes, and additional experiments will be required to clarify this. Nonetheless, together with the *shi^ts^* rescue, this result suggests that involvement of postsynaptic partners is required to initiate R7 axon degeneration. It further suggests that prolonged hyperpolarization of the postsynaptic neurons represents an important signal for the initiation of axonal degeneration in R7.

### Synapse loss precedes axonal degeneration

**Figure 9.**
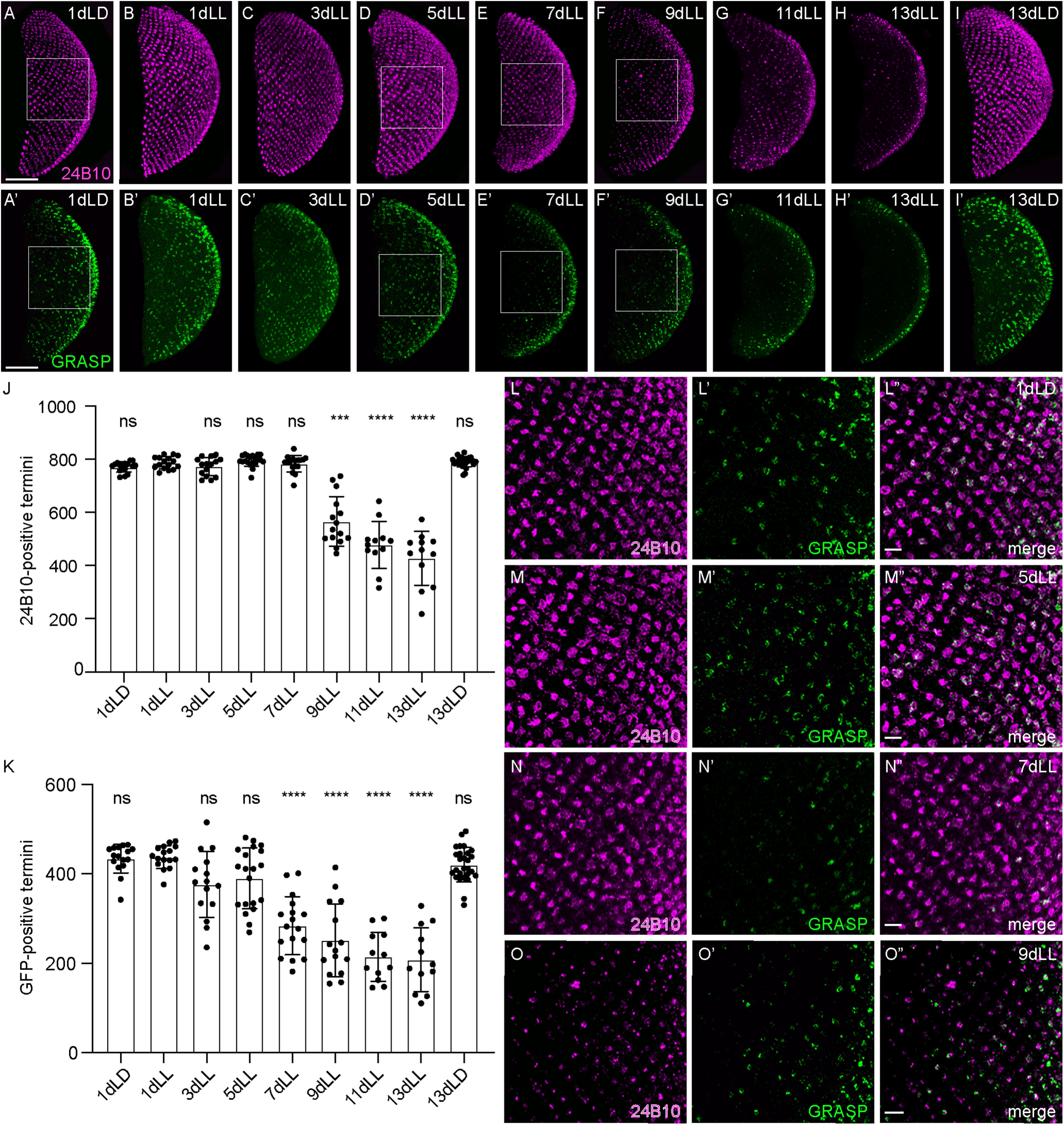
Synaptic dysfunction between R7 and Dm8 precedes axonal degeneration. ***A–I'***, Axonal termini of R7 in the medulla of *w/GMRwhite RNAi; Rh4-LexA/LexAop-spGFP1-10, UAS-CD4spGFP11; Ort-C2-Gal4/*+ flies exposed for various time to LL or control conditions (LD). ***A–I***, Axon termini labeled with chaoptin (24B10) in magenta. ***A'–I'***, depict GFP-positive termini in which a GRASP event between R7 and Dm8 took place (green). Squares highlight regions shown in close ups (***L–O”***). ***J***, 24B10-positive R7 termini in the medulla of flies exposed for various time to LL or control LD conditions. ***K***, GRASP-positive R7 termini numbers in the same medullas as in ***J***. Error bars indicate mean ± SD (ns, not significant, *** *P* < 0.001, ^,****^ *P* < 0.0001). ***L–O”***, Close-ups on the axonal termini of R7 in the medulla of flies exposed for various times to constant light (5 d in ***M–M”***; 7 d in ***N–N”***; 9 d in ***O–O”***) or to control condition for 1 d in LD (***L–L”***). ***L–O***, Termini stained with chaoptin (24B10) in magenta. ***L'–O'***, Depiction of sybGRASP-positive contacts between R7 and Dm8 (green) and a merge of the close-ups for 24B10 and GFP. Scale bar, 5 µm.

**Figure 10.**
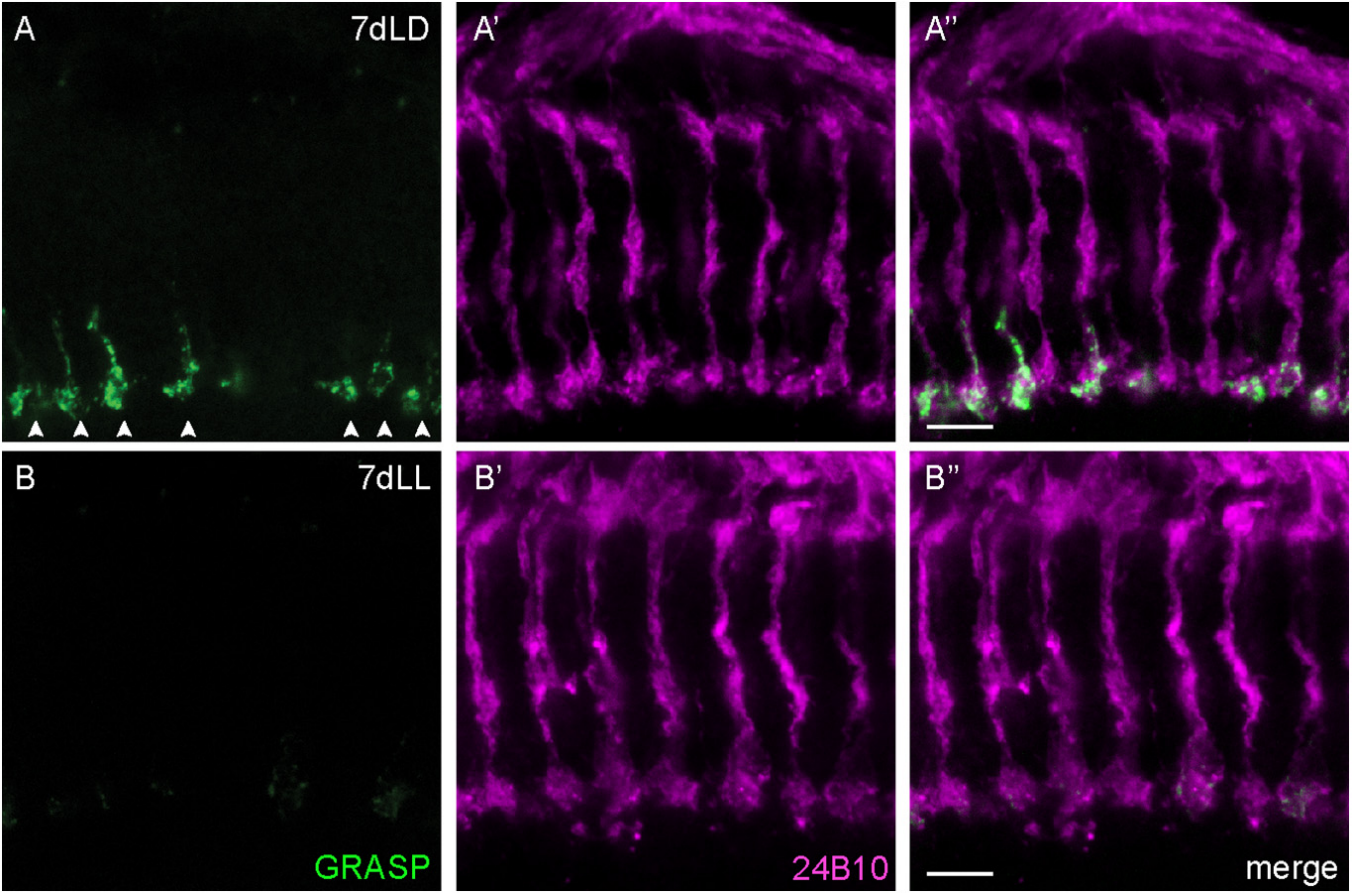
Axonal integrity of R7 is preserved upon loss of its synaptic function. ***A–A”***, ***B–B”***, R7 axons in the medulla imaged in a dorsal to ventral orientation showing the presence of GRASP-positive synapses (green in ***A***, ***A***,”; arrowheads in ***A***) and intact axons labeled with chaoptin (24B10) in magenta (***A'***, ***A”***) in a 7dLD control condition. Upon constant light exposure, the GRASP signal disappears in axonal termini after 7 d (green in ***B***, ***B”***), whereas intact axons are still present (24B10, magenta in ***B'***, ***B”***). Scale bars, 5 µm.

Synaptic dysfunction is considered as an early event and as the major determinant of ND, including AD and PD (Bellucci et al., 2016; Colom-Cadena et al., 2020; Compta and Revesz, 2021). We thus asked whether synaptic function was also affected in our model. For this, we took advantage of the activity-dependent syb:GRASP system, which allows for retrospective labeling of synapses based on their activity (Macpherson et al., 2015). We expressed the *syb-spGFP1–10* construct in the *yellow* ommatidia subset of R7s using *Rh4lexA* as a driver and *CD4-spGFP11* in Dm8, a synaptic partner of R7 in the medulla, using *ortGal4* (Gao et al., 2008; Schnaitmann et al., 2018). Only the combination of the two fragments across the plasma membrane of active synapses yields a detectable GFP signal (Macpherson et al., 2015). These flies were then subjected to a control LD treatment (1 d and 13 d) or put in LL for different periods of time (1, 5, 7, 9, 11, or 13 d). At every time point, 24B10-positive axonal termini were counted (Fig. 9*J*) and compared with numbers of GFP-positive termini, which represent active synapses between the *yellow* R7 PRCs and Dm8 (Fig. 9*K*). We counted a total number of ∼750 R7 axonal termini labeled with 24B10 per medulla (Fig. 9*J*) as well as ∼450 GRASP-positive termini (Fig. 9*K*) in control conditions (1dLD), corresponding to the approximate expected fraction of *yellow* ommatidia (70% of total R7; Franceschini et al., 1981). These numbers remained constant over time in an LD cycle (Fig. 9*J*,*K*). In this genotype, 24B10-positive termini were unchanged at 7dLL (Fig. 9*E*,*J*,*N*,*N”*), whereas axonal degeneration started at 9dLL (Fig. 9*F*,*J*,*O*,*O”*) and proceeded progressively in the following days (Fig. 9*J*). In contrast, GRASP counts were already clearly reduced at 7 d in LL (drop from ∼450 to ∼300 GFP-positive termini; Fig. 9*E'*,*K*,*N'*,*N”*), thus before axonal degeneration was detectable. We confirmed axon integrity imaging the medulla in a dorsal to ventral orientation (7dLD; Fig. 10*A–A”*). In these images, loss of GRASP signal (Fig. 10*B*,*B”*) contrasted with the intact appearance of axons (24B10) at this time (Fig. 10*B*',*B”*).

Together, these data indicate that activity-dependent synaptic transmission between R7 and a medullar postsynaptic partner is lost before axonal termini numbers start to decrease, suggesting that loss of synaptic connection precedes axonal degeneration. Loss of synaptic connections could thus represent an initial step toward degeneration. Alternatively, loss of synaptic components could yield reduced synaptic transmission to preserve circuit homeostasis and thus be initially protective at early stages of prolonged activation (Sugie et al., 2015). Thus, the model system of sporadic axonal degeneration that we developed shares important characteristics with the reported progression of neurodegeneration in human ND.

## Discussion

In this work, we established a model of sporadic initiation of axonal degeneration. We describe here the amenability of using R7 photoreceptor axonal termini numbers in the medulla as a readout for sporadic axonal degeneration. For this, we developed tools for the quantitative analysis of R7 axon loss in the medulla. With this system, we defined that the stage of initiation of axonal degeneration is reached individually for each neuron. Furthermore, we showed that synapses between R7 and a postsynaptic partner are dysfunctional before axonal degeneration starts. We thus produced a model system in which we recapitulate important early features of human neurodegenerative diseases. In addition, we provided initial evidence for a postsynaptic signal regulating stability of the presynaptic axon. Thus, the model we established can now be used to define the early changes that happen within neurons at the time point in which their resilience capacity is overcome.

Various factors can affect axon vulnerability, including cell senescence, metabolic changes, neuroinflammation and exposure to chemicals, including some used in cancer therapy (Coleman, 2005; Adalbert and Coleman, 2013; Neukomm and Freeman, 2014; Figley and DiAntonio, 2020). In this model, we induced axonal degeneration by stimulating neurons for prolonged periods of time. Photoreceptors are resistant to prolonged stimulation, thanks to a series of mechanisms acting on a short time scale to maintain their response and output within a dynamic range and potentially to maintain circuit homeostasis on a longer time scale (Stavenga, 1995; Juusola and Hardie, 2001; Sugie et al., 2015). Thus, they represent a good system to investigate how this resilience is no longer sufficient to guarantee axon maintenance. Further, imbalanced activity represents a potential trigger of neurodegeneration (Palop et al., 2007; Busche and Konnerth, 2016; Palop and Mucke, 2016; Sosulina et al., 2021). Neuronal hyperactivity has been detected in patients with mild cognitive impairment, a prodromal stage of AD, and in carriers of the *APOE4* allele, the most important genetic risk factor for late-onset sporadic AD, as well as in many transgenic AD mice (Hector and Brouillette, 2020). Furthermore, activity imbalance at the circuit level is reported in AD and in AD model systems and is considered a potential trigger of neurodegeneration (Palop et al., 2007; Busche and Konnerth, 2016; Palop and Mucke, 2016; Sosulina et al., 2021). Although more specific experiments will be required, our present data suggest that an unbalanced level of activity within the local microcircuit at the PRC output synapses is at the core of the transition toward initiation of axonal degeneration in this system. The signals that trigger the onset of degeneration remain to be identified and could be counterbalancing pathways that initially maintain potentially protective homeostasis (Sugie et al., 2015; Orr et al., 2020).

Axonal degeneration induced in constant light conditions does not affect a particular region of the medulla. This stochastic start of degeneration is preceded by the swelling of R7 axonal termini, which takes place throughout the medulla. This change appears similar to that reported in degenerating axons in the CNS of rodents (Ertürk et al., 2007).

The swelling is reversed by placing the animals back in the dark for prolonged periods of time. In contrast to the swelling, which was shared by all axons, only individual axons lost their tubulin and membrane markers and degenerated. At 7dLL, the first axons started degenerating, but putting 7dLL animals back in DD blocked further axonal loss, indicating that the timing of trigger rather than the timing of execution of an axonal degeneration program is different among PRCs. This suggests that the resilience capacity of a single axon is different from that of its neighbors, and it will be crucial to understand the mechanisms underlying this phenomenon.

We observed that synapses between R7 and one of its postsynaptic partners in the medulla lose their integrity before axons degenerate. These results raise the question of whether synaptic detachment could be causative of axonal degeneration or whether synaptic maintenance mechanisms were lost before synapses detached. A broad line of evidence suggests that alterations in synaptic adhesion play key roles in the disruption of neuronal networks in neurodegenerative diseases (Chapman, 2014; Leshchyns'ka and Sytnyk, 2016; Kilinc, 2018). Synaptic maintenance needs to cope with the metabolic demand of neurotransmission, as well as with elevated rates of protein turnover and a high membrane exchange that requires efficient delivery and constant supply of newly synthesized proteins (Harris et al., 2012; Guedes-Dias and Holzbaur, 2019). Therefore, removal of damaged proteins and organelles from synaptic sites is essential to sustain synaptic function (Andres-Alonso et al., 2021). Abnormalities in function, trafficking, or signaling of mitochondria, lysosomes, and endoplasmic reticulum contribute to development of neurodegenerative diseases (Kerr et al., 2017; Cabral-Miranda and Hetz, 2018; Lie and Nixon, 2019; Öztürk et al., 2020). It will thus be of high interest to clarify whether axonal transport deficiencies, ER stress, or mitochondrial and/or lysosomal dysfunction precede synaptic detachment in this model.

We found no signs of apoptosis in R7 upon up to 13 d of exposure to constant light, even when axonal degeneration was very advanced. Thus, in this model, axonal degeneration precedes cell death by several days, reproducing the sequence of events observed in human neurodegenerative diseases (Coleman, 2005). Interestingly, we observed axonal swellings and microtubule disassembly upon light treatment, both features often preceding cell body loss in the neurite retrograde degeneration (dying back pathology) described in many neurodegenerative diseases (Wang et al., 2012). Increasing evidence shows that injury-induced degeneration (Wallerian degeneration) shares molecular features with ND dying-back axonal degeneration; the local loss of NMNAT2 activates either SARM1 Wallerian degeneration or triggers SARM1-dependent dying back (Yaron and Schuldiner, 2016; Neukomm et al., 2017; Coleman and Höke, 2020; Figley and DiAntonio, 2020). For this reason, it would be crucial to address the involvement of NMNAT2, SARM1, and other players of this pathway in our model.

Together, we developed a quantitative stochastic model of axonal degeneration. Our initial characterization indicates that it reproduces important traits of human neurodegenerative diseases, including the interruption of neuronal resilience to repetitive activation and the limitation of the initial defects to the axons, without resulting in immediate cell death. Our results further point to a role of circuit imbalance toward the initiation of axonal degeneration.
